# Effects of Bifidobacteria Fermentation on Physico-Chemical, Thermal and Structural Properties of Wheat Starch

**DOI:** 10.3390/foods11172585

**Published:** 2022-08-26

**Authors:** Jing Hong, Wanxue Guo, Peixia Chen, Chong Liu, Juan Wei, Xueling Zheng, Saeed Hamid Saeed Omer

**Affiliations:** College of Food Science and Engineering, Henan University of Technology, Zhengzhou 450001, China

**Keywords:** wheat starch, bifidobacteria, fermentation, pasting property, thermal stability

## Abstract

Lactic acid bacteria have been considered to be a very important species during sourdough fermentation. In order to explore the effects of bifidobacteria fermentation on thermal, physico-chemical and structural properties of wheat starch during dough fermentation, starch granules were separated from the fermented dough at different fermentation times, including 0 h, 2 h, 6 h, 9 h and 12 h. The results showed that the morphology of starch granules was destroyed gradually as the fermentation time increased, which appeared as erosion and rupture. With the increase in fermentation time, the solubility showed a significant increase, which changed from 8.51% (0 h) to 9.80% (12 h), and the swelling power was also increased from 9.31% (0 h) to 10.54% (12 h). As for the gelatinization property, the enthalpy was increased from 6.77 J/g (0 h) to 7.56 J/g (12 h), indicating a more stable thermal property of fermented starch, especially for the longer fermentation. The setback value was decreased with short fermentation time, indicating that the starch with a longer fermentation time was difficult to retrograde. The hardness of the gel texture was decreased significantly from 50.11 g to 38.66 g after fermentation for 12 h. The results show that bifidobacteria fermentation is an effective biological modification method of wheat starch for further applications.

## 1. Introduction

Starch-based food is the most consumed food in the world and is an especially important part of the staple food of the oriental table [1]. It is rich in nutrition and is the main source of daily calorie intake of human beings [2]. Wheat flour is the main ingredient in steamed bread, bread, pasta and other starch-based foods, and its starch content accounts for 78–82% [3]. There are many problems in the quality of starch-based foods, which may be due to differences in raw materials or processes, such as small specific volume, high hardness, easy aging and insufficient flavor of bread and steamed bread. Rice flour and noodles have insufficient elasticity, poor chewiness and an unsmooth taste. Therefore, it is important to improve the quality of starch-based food through the improvement of the processing technology. Studies have shown that when sodium alginate is added to wheat flour for bread making, it can increase the baking rate of bread, as well as the swelling of bread dough [4]. It was found that extracellular polysaccharides produced by lactic acid bacteria (LAB) during fermentation improved the quality of bread more than the addition of extracellular polysaccharides alone [5]. Although additives can improve the quality of starch foods, consumers prefer natural and healthy foods produced by microbial fermentation and other technologies.

Sourdough fermentation is one of the oldest biotechnologies used in the production of cereal foods, which can improve the quality of flour products. Up to now, many research works have been focused on the improvement of flour products’ quality through sourdough fermentation [6,7,8,9,10]. Sourdough fermentation can increase the specific volume [6], improve texture and nutritional value [7,8], enhance flavor [9] and prolong the shelf life of products [10]. LAB are the main species of sourdough fermentation, and the fermented starch-based food not only has good quality but also high safety. Fermentation could change the multi-scale structure and gelatinization characteristics of wheat flour, leading to the improvement of the quality of starch-based foods [3]. LAB fermentation can modify starch particles, make a dough with stronger viscosity, softness and less elasticity, and improve the ability to withstand the pressure of expanding carbon dioxide to obtain a better quality of corn bread [7]. In the fermentation process, microorganisms can use carbohydrates to produce gas, acid and enzyme, thus changing the structure of starch and making the structure of starch products loose and porous, along with good flavor and aroma [11]. Fermentation can actively improve the nutritional quality of whole wheat products by delaying starch digestibility [12].

The fermentation process is different from direct treatment with acid or enzymes. Due to the continuous process of fermentation, the degradation of starch chains caused by α-glucoamylase, which is produced/activated during the fermentation process, may proceed gradually during fermentation, resulting in the positive improvement of starch properties, such as the enhancement of thermal properties [13]. As a ubiquitous probiotic in the human body, Lactobacillus and Bifidobacterium are commonly used as bacteria starters in sourdough fermentation. Some researchers consider bifidobacteria as LAB and as beneficial during sourdough fermentation [14,15]. Others think it is a different kind of bacteria [16,17]. No matter how they are classified, there is a consensus that bifidobacteria make a significant contribution to texture during fermentation. Bifidobacteria are a type of probiotic bacteria that produce organic acid and are beneficial to human health [18]. Bifidobacterium can synthesize and produce vitamins, such as riboflavin, thiamine, vitamin B6 and vitamin K, and related bioactive molecules, such as folic acid, nicotinic acid and pyridoxine [19]. It can also show a good adaptability to the dough ecosystem and contributes to acidification, which can significantly reduce the phytate content in whole wheat bread [20]. The number of dietary products containing bifidobacterium has increased significantly [21]. So far, bifidobacterium has already attracted more and more attention due to its anti-bacteria, anti-cancer properties, improving immunity, nutrition and health care. Due to its essential contribution and unclear influence on the polysaccharides of starch granules in the sourdough fermentation, it is necessary to explore the effect of bifidobacteria on the properties of starch isolated from fermented dough.

## 2. Materials and Methods

### 2.1. Materials

Wheat flour (13.81% moisture content, 10.70% protein content, 70.40% starch content) was purchased from a local supermarket (YIjiayi flour, Yijiayi natural flour Co., Ltd., Xuchang, China); Bifidobacteria powder (10^10^ CFU/g) was commercially available.

### 2.2. Preparation of Wheat Starch Separated from Fermented Dough

Amounts of 100 mL water and 2 g bifidobacteria powder (based on 1% of flour weight) were added to 200 g (db) flour. The mixture was fully stirred in a mixing machine (SZM-10, Guangzhou Xuzhong Food Machinery Co., Ltd., Guangzhou, China) for 4 min. Then, the dough was fermented for 0 h, 2 h, 6 h, 9 h and 12 h, respectively, at a temperature of 35 °C and relative humidity of 85%. The fermented dough was taken out and then washed with distilled water until the washing liquid was clear with non-cloudy starch slurry. After the starch slurry was centrifuged at 1509.3× *g* for 15 min, the supernatant was discarded. The protein impurities in the upper layer were scraped carefully, and the starch in the lower layer was collected. After freezing and drying, the starch was ground and passed through a 100-mesh sieve.

### 2.3. Determination of Damaged Starch Content

The content of damaged starch was determined with the UCDc value using Chopin’s SD matic damaged starch meter (SD matic, Chopin Technologies, Paris, France). The content of damaged starch can be obtained by calculating the absorption rate of iodine, and the results can be displayed by UCD (Chopin Dubois Unit) and UCDc. UCDc is the UCD corrected by water and protein content. UCD = 0.95·AACC + 10.17. Thus, UCD can also be converted into the AACC expression by using the unit of %. The experiment for each group was repeated three times.

### 2.4. Morphological Properties

A Quanta 250 FEG model scanning electron microscope (FEI INSPECT F50, FEI, Hillsboro, OR, USA) was used to investigate changes that occurred on the granular surface at different fermentation times. The starch samples were sprayed onto a double-sided adhesive tape coated with a thin gold layer and then fixed on the sample table. The starch granules were examined at an acceleration voltage of 10 kV.

### 2.5. Solubility and Swelling Power

The solubility (*S*) and swelling power (*SP*) of starch were determined according to the method of Liu et al. [22]. Amounts of 1 g (db) starch and 49 mL distilled water were mixed in a centrifugal tube to obtain 2% slurry. Each sample was mixed with distilled water to obtain the starch suspension, which was then stirred in a water bath at 90 °C for 30 min. After cooling to room temperature (25 °C), the samples were centrifuged at 1509.3× *g* for 15 min to collect the supernatant. The liquid supernatant was dried in a drying oven (FXB101-2, Shanghai Shuli Instrument Co., Ltd., Shanghai, China) at 105 °C to a constant weight. Solubility was expressed as the percentage of dry solid weight based on the weight of the dried sample. The swelling power was calculated as the weight ratio of the wet sediment to the initial dry matter, excluding the water-soluble starch. The experiment for each group was repeated three times. The solubility (*S*) and swelling power (*SP*) of starch were calculated according to the following formula, and the average value of the two measurement results was calculated in Equation (1).

(1) $$\begin{array}{l} S/\%=\frac{B}{M}\times 100\\ SP/\%=\frac{C}{M\times \left(100-S\right)}\times 100 \end{array}$$

*B*—the weight of water-soluble starch, with unit of g; *C*—the weight of swollen gel, with unit of g; *M*—initial dry matter, with unit of g.

### 2.6. X-ray Diffraction (XRD)

The X-ray diffraction patterns of starch samples were determined using a Bruker-AXS Model D8 Advance diffract meter (Model D8, Bruker AXS GmbH, Karlsruhe, Germany) under the following conditions: Cu-Kα radiation (λ = 1.543), Nickel filter, 40 kV and 30 mA. The intensities were measured in the 10–30° diffraction angle range with a 0.03° step size and measuring speed of 4°/min. The experiment for each group was repeated twice. The relative crystallinity was calculated as the ratio of the intensity of diffraction peak to the total intensity.

### 2.7. Thermal Properties

The thermal properties of starch samples were determined by using a differential scanning calorimeter (DSC-7, Perkin-Elmer, Waltham, MA, USA). A 2.5 mg (db, dry basis) starch sample was placed in an aluminum case, and distilled water was added to obtain a starch/water ratio of 1:3 by weight. The aluminum case was sealed and equilibrated at room temperature for 24 h. Then, it was heated from 20 to 120 °C using a scanning rate of 10 °C /min. A sealed empty case was used as a reference. The experiment for each group was repeated three times. The characteristic temperatures of the transitions were recorded as the onset temperature (T_0_), peak temperature (T_P_), conclusion temperature (T_C_) and enthalpy of gelatinization.

### 2.8. Pasting Properties

The pasting properties of starch samples were studied using a Rapid Visco-Analyzer (RVA series 3, Newport Scientific Instruments, Narrabeen, Australia). About 3 g starch samples (corrected to 14% wet basis) were mixed with 25 mL water and then added to the RVA canister for testing. A programed heating and cooling cycle was used at a constant shear rate, where the starch samples were held at 50 °C for 1 min, heated from 50 °C to 95 °C at 9 °C/min, held at 95 °C for 3 min, cooled to 50 °C at 9 °C/min and held at 50 °C for 5 min. The experiment for each group was repeated three times.

### 2.9. Gel Textural Properties

After RVA determination, the sample in the reaction cup was placed in a weighing bottle for 24 h at room temperature to form the gel. The texture profile analysis (TPA) of the starch gel was then evaluated by the texture analyzer (TA XT, Newport Scientific Instruments, Narrabeen, Australia). A cylindrical probe P/6 was used for the test (pre-test speed 2.0 mm/s, test and post-test speed 1.0 mm/s, with 5 g trigger force at a distance of 2.0 mm). The time interval between the two measurements was 3.0 s. The gel texture properties, including hardness, adhesiveness, springiness, cohesiveness, gumminess and resilience, were recorded. The experiment for each group was repeated three times.

### 2.10. Statistical Analysis

For the statistical analysis of the data in this work, Excel and SPSS 20.0 software were used, while the ORIGIN 8.0 software program was used for drawing. The experiment for each group was repeated three times, except for the results of SEM (once, with the representative vision) and XRD (twice). The results were expressed as the mean standard deviation. The data were analyzed by the analysis of variance (ANOVA), and significance was established at *p* < 0.05.

## 3. Results and Discussion

### 3.1. Damaged Starch Content during Bifidobacteria Fermentation

**Figure 1:**
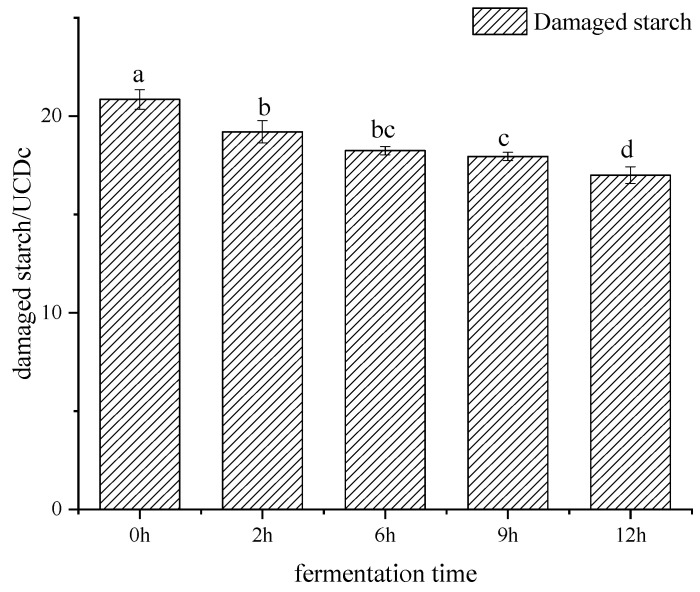
Damaged starch content of starch separated from bifidobacteria-fermented dough under different fermentation times. The representation of different letters have significant difference (*p* < 0.05).

The purity of total starch content for each sample was about 97–98%, according to the method of the total starch assay kit, following the approved protocols from Megazyme. Due to the external action, the internal structure and external shape of starch were damaged, even with the appearance of cracks and fragments, which was considered damaged starch. As shown in Figure 1, the damaged starch content was decreased significantly from 0 h to 12 h. The damaged starch content was the lowest after 12 h of fermentation, with the value of 17 UCDc. Studies have shown that the second source of fermentable sugars is damaged starch, which can be degraded into maltose by amylase [23]. During dough fermentation, the α-amylase in flour is activated to degrade the damaged starch, which provides the energy for microbial growth. Therefore, the content of damaged starch decreases significantly with the increase in fermentation time.

### 3.2. Morphological Property

**Figure 2:**
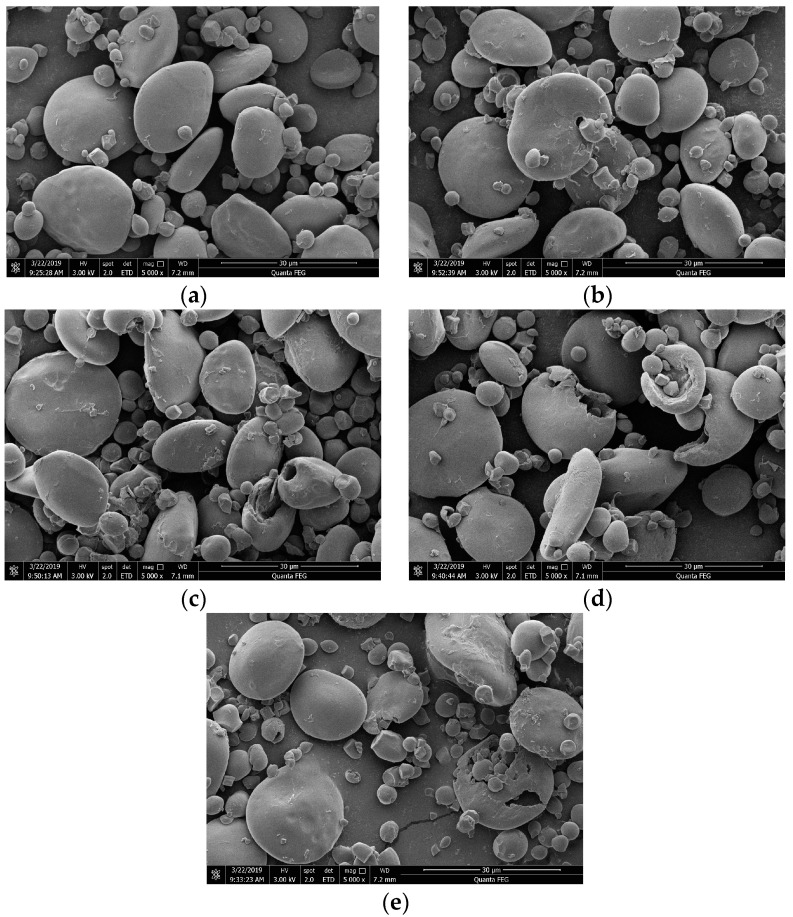
Scanning electron microphotographs of starches separated from bifidobacteria-fermented dough at different fermentation times: (**a**) 0 h, (**b**) 2 h, (**c**) 6 h, (**d**) 9 h, (**e**) 12 h.

The surface morphology, size and external characteristics of starch granules can be observed to provide effective information [24]. SEM was used to obtain the visual evidence to show the changes in the morphology of wheat starch granules during the fermentation of bifidobacteria. As shown in Figure 2, the native starch granules were intact and appeared as a disk shape of the A-type starch granules with a diameter of 10–35 μm, while the spherical shape was defined as B-type starch granules with a diameter of 2–8 μm, which were identified in a study by Jane et al. [25]. As seen in Figure 2a (0 h), the surface of the native starch was smooth, and the shape was intact, without obvious erosion. After the fermentation of bifidobacteria, it was greatly destroyed, as seen in Figure 2b–e. For example, the obviously corroded voids were observed at 2 h and 6 h fermentation, and heavier and larger damage occurred in starch granules with the fermentation time increased to 9 h and 12 h (Figure 2d,e). This indicated that the starch granules can be eroded and damaged during dough fermentation. More serious damage was observed when the fermentation time was prolonged, which might be caused by the increase in organic acid produced by bifidobacteria. In addition, it can also be speculated that the bifidobacteria can obtain their carbon source from damaged starch, which was produced as a result of extensive damage to the surface of the starch, as proved by Liu et al. [26]. Mahsa et al. [27] found that organic acids can cause cracks in starch granules. In addition to that, it was interesting to note that the damage seemed to be more evident in large A-type starch granules in comparison with B-type starch granules. A similar finding was reported by Zhao et al. [3], who pointed out some larger and deeper cracks were found after a long period of LAB fermentation on wheat starch, especially for large A-type granules. 

Chinsamran et al. [28] also found that starch granules of cassava, sweet potato and rice starch remained intact, but their surfaces became rough after fermentation. Moreover, the granular surface became more irregular after LAB fermentation compared with natural fermentation. Natural fermentation is a process in which microorganisms in the natural environment are used for fermentation. Similarly, the wheat starch granules in this study also showed more severe damage after bifidobacteria fermentation (Figure 2a,d) when compared with nature fermentation [3]. However, different results were obtained by some researchers [29,30]. They concluded that starch granules isolated from fonio were swelled and clumped after yeast and LAB fermentation, which may be attributed to the leaching and swelling of starch granules caused by the enzymatic action of microorganisms. The different phenomenon observed in the surface morphology of starch granules may be related to the different starch sources and fermentation methods.

### 3.3. Solubility and Swelling Power

Due to the strong hydrogen bonds, intact starch granules are insoluble in cold water but can absorb water and swell to form a paste solution when being heated in the presence of water upon gelatinization temperature [31]. Partial starch granules can dissolve in water, which is mainly caused by the destruction of the starch crystalline structure as well as the hydroxyl groups’ interaction between the water molecules and amylose and amylopectin molecules. In this process, the solubility and swelling power are commonly used to reflect the interaction between starch and water, which have important effects on the processing characteristics of starch-based foods. 

**Figure 3:**
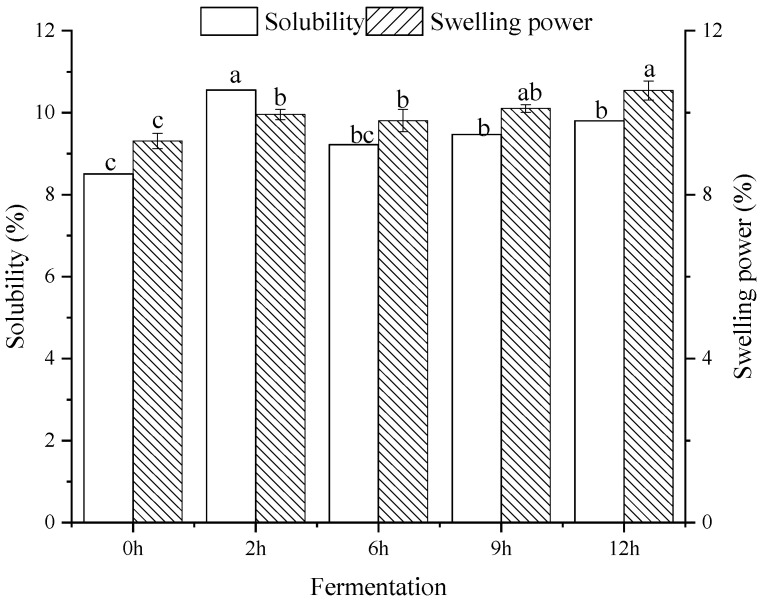
Solubility and swelling power of starch samples separated from bifidobacteria-fermented dough at different fermentation times. The representation of different letters have significant difference (*p* < 0.05).

The solubility and swelling power of starch separated from bifidobacteria-fermented dough are shown in Figure 3. The solubility was increased from 8.51 to 9.80% during the initial fermentation stage (from 0 h to 2 h) and reached a maximum value at 2 h. Then, it was decreased after being fermented for 6 h, which was higher than native starch, and remained at a relatively constant value as the fermentation proceeded. Sanz et al. [32] pointed out that bifidobacteria fermentation can produce lactic acid and acetic acid to reduce the pH value of dough, thus inducing the increased solubility by amylose leaching, which might be caused by the acidic hydrolysis of starch granules and the destroyed structure [33]. On the other hand, the metabolite lipase produced during the fermentation process acted on the substances of fat, which may improve the rate of starch granules’ water absorption, resulting in increased solubility [34]. After fermentation for 6 h, the solubility decreased significantly. More organic acids and enzymes produced by fermentation with a prolonged fermentation time were responsible for this phenomenon. 

As Figure 3 shows, the swelling power showed a significant increase from 9.31% (0 h) to 10.54% (12 h) with an increase in fermentation time. This may be related to the debranching of amylopectin or partial hydrolysis of the side chain during fermentation. The amylose content may be reduced during fermentation [35], and the lower content of amylose is the main reason for the larger swelling power [36]. It was found that the capacity of starch granules to swell was related to the internal structure of the semi-crystalline region, which was composed of the alternating arrangement of amylose and amylopectin; thus, the amylose/amylopectin ratio has been considered an important factor in controlling the swelling behavior of the starch [34]. Furthermore, increased swelling power was correlated with increased fermentation time, indicating that the destruction of the starch structure in the fermentation process has a direct relationship with the increase in fermentation time [37], which has also been confirmed by SEM images in this study. In general, fermentation can destroy the starch structure, thus resulting in a higher hydration and continuous expansion of the starch granules during heating, thus producing a higher expansion force [38].

### 3.4. Changes in Crystallinity during Fermentation

**Figure 4:**
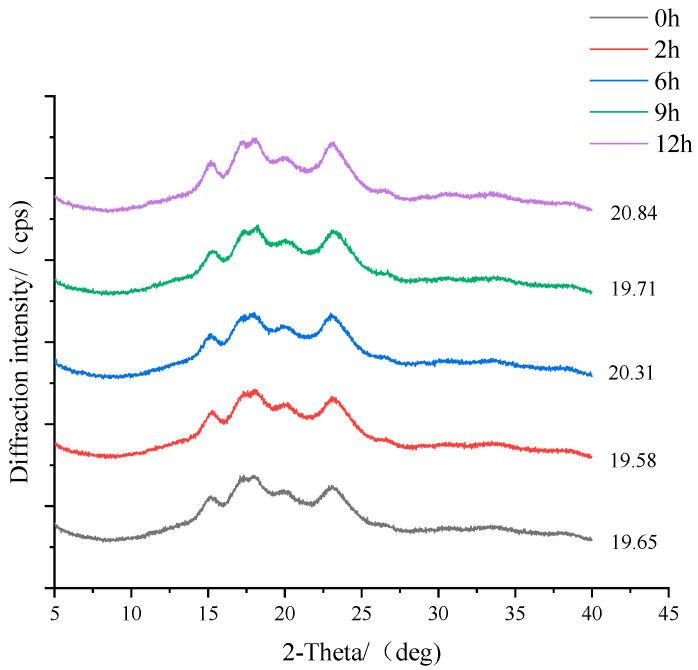
X-ray diffraction patterns for starch samples separated from bifidobacteria-fermented dough at different fermentation times.

X-ray diffraction was used to determine the long-range structure of starch granules. The X-ray diffraction patterns of wheat starch samples with various fermentation times are presented in Figure 4. It is well known that starch granules have the characteristics of a semi-crystalline structure in which the crystallite and the amorphous region appear alternately [39]. The amorphous region is mainly composed of amylose and a small amount of amylopectin. The diffraction peaks on the spectrum can reflect the crystalline regions within the starch granules. As illustrated in Figure 4, the diffraction peaks occurring at 2θ = 15°, 17°, 18° and 23°, as well as the amorphous region of dispersive peaks, suggest that the structure of wheat starch is a typical A-type pattern [25]. A small peak was also found at 20°, which represents the V-type crystalline structure in B-granules as a result of protein, fat and fiber components present in them [40]. As shown in Figure 4, a stronger diffraction at 20° indicates that the formation of amylose–lipid complexation is promoted by fermentation. The fermented wheat starch showed the same XRD pattern as the native starch, even though the diffraction intensity was changed, implying that the starch crystalline structure was not altered by bifidobacteria fermentation. Although changes were found in the morphology of starch granules, they were not sufficient to change the crystalline type. The results were also proven by previous research [13]. However, different findings were also reported by Camargo et al. [41], who found that the diffraction mode of native cassava starch was changed from C type to A type when treated with the organic acid directly. This evolution of the crystalline structure was supposed to be achieved through the removal of constraints exerted by the amorphous region [42]. Hence, we considered that the acid hydrolysis behavior of starch during bifidobacteria fermentation was different from direct treatment with acid, as well as the different starch sources.

The change in the relative crystallinity is also presented in Figure 4. The crystallinity of native starch was 19.65% and slightly increased to 20.84% after 12 h fermentation, which indicates that the structure of the starch granules was weakly destroyed. This change mainly occurred in the amorphous regions, which were weakened or destroyed, resulting in a greater relative crystallinity [43]. Similar results were also reported by other researchers [3,43] who considered the higher crystallinity was also associated with the improvement of the double helix sequence of amylopectin molecules.

### 3.5. Thermal Properties

The DSC results of wheat starch at different fermentation times are shown in Table 1, which include the onset temperature (To), peak temperature (T_P_) and conclusion temperature (T_P_) of gelatinization, as well as the gelatinization temperature range (Tc-To) and enthalpy (∆H). Starch gelatinization refers to the first-phase transition from the semi-crystalline state to the amorphous state in the presence of water and temperature [44]. The thermal properties of starch can be affected by many factors, including the morphology, size and structure of the starch granules, the ratio of crystalline area to the amorphous area and the fermentation conditions [45]. Studies have shown that the gelatinization enthalpy is positively correlated with the crystallinity and particle size of the starch granules [43]. It was observed that bifidobacteria fermentation has no significant influence on To, Tc and Tc-To of starch samples. However, the fermented starch samples had a higher T_P_ compared with native starch, and there was no significant difference between the samples with different fermentation times. It was obvious that the increase in T_p_ might be due to the destruction of the starch structure by the enzyme or acid hydrolysis produced during the fermentation. Chinsamran et al. [28] pointed out that the less ordered starch molecules in the amorphous region were more sensitive to acid hydrolysis, leading to the crystallites decoupling. As a result, the crystalline area was no longer destabilized, and the gelatinization temperature was increased consequently. Alonso et al. [45] also indicated that the increased T_P_ during fermentation may be related to the increased crystallinity of starch. 

Gelatinization enthalpy was increased during the initial stage of fermentation (from 0 to 6 h) and reached a maximum at 6 h. Then, it was decreased slightly but was still higher than that of native starch, which was attributed to the preferential hydrolysis of the amorphous regions and the improvement of the double helix order, as observed in XRD. A similar result was found in the hydrolysis of corn starch by direct treatment with acid [43]. In addition, the research found a significantly positive correlation between gelatinization enthalpy and relative crystallinity. However, the change of crystallinity did not present such a trend in our experiment. The same effect was also observed by Alonso et al. [45] who found that the gelatinization enthalpy was changed by fermentation, and there was no correlation between enthalpy and fermentation time.

### 3.6. Pasting Properties

Starch granules began to swell and break, forming a uniform paste solution when heated in water. As shown in Table 2 of the RVA test, peak viscosity, through the value and final viscosity of starch samples, showed a significant decrease during the initial stage of fermentation (from 0 to 2 h) from 2247 cP to 2033 cP, 1769.5 cP to 1512.5 cP and 2816.5 cP to 2221 cP, respectively. The samples reached a minimum at 2 h and were increased after fermentation for 6 h, then remaining relatively constant as the fermentation proceeded. Similar observations were reported in peak viscosity for the fermented cassava flour starch [46] and soybean flour [47]. Li et al. [48] pointed out that amylopectin with a high branching degree has a strong ability to absorb and retain water molecules, resulting in higher peak viscosity. Therefore, we believe that amylopectin was degraded during fermentation, and its branching degree was decreased. Widya et al. [49] also considered the decrease in peak viscosity was due to the decreased molecular weight of amylose and amylopectin after being attacked by the acid and enzyme, which also confirms the reason for the high solubility of modified starch. The changed viscosity has also been reported by other researchers [50,51], which demonstrated that the decrease in pasting viscosity after fermentation was attributed to the higher solubility of starch caused by the production of short chains during fermentation. It was assumed that the increase in viscosity at 6 h fermentation may be related to the change in solubility in this study, and pasting viscosity was negatively correlated with solubility.

The setback value reveals the ability of amylose to form gel during the cooling process of starch paste and reflects the retrogradation degree as well [52]. As shown in Table 2, the setback value of samples fermented for 0 h was 1047 cP, and it changed to 707 cP, 887 cP, 734 cP for 2 h, 9 h and 12 h, respectively. The setback values of fermented samples were significantly lower than those of unfermented samples. This indicated that the fermentation process of bifidobacteria could significantly improve the retrogradation characteristics of wheat starch. Yang et al. [11] found that the retrogradation of rice starch was greatly inhibited by the reduced pH value. Similarly, the organic acid yielded during bifidobacterial fermentation could also decrease the pH value in this study. Olanipekun et al. [37] also found that the setback value was observed to decrease with a prolonged fermentation period. This suggests that the tendency was retrograded and decreased by fermentation.

### 3.7. Gel Textural Properties

After gelatinization, the starch molecules will rearrange to form a gel network during cooling. Texture profile analysis can simulate the human chewing process and evaluate the textural characteristics [24]. As can be seen from Table 3, the hardness values remained stable during the initial stage of fermentation (from 0 to 2 h). However, they showed a significant downward trend with the increase in fermentation time, while the hardness of the starch gel decreased from 50.1 g to the lowest value of 38.7 g after being fermented for 12 h, which indicated that the softness of the gel was increased after bifidobacteria fermentation. Chinsamran et al. [28] also found the hardness of sweet potato starch gel decreased significantly after natural fermentation. A similar result has also been observed by Festus et al. [53]. It showed the hardness of cassava flour gel was reduced by both natural and cultured fermentation. Gel hardness has been reported to be related to the network formed by the leached amylose, as well as the swelling degree of the starch granules, which is mainly related to the high affinity of amylose when forming the connected double helix aggregates [27,53]. The hardness of the gel was reduced as a result of the changed network caused by fermentation.

It was also noticed that the adhesiveness and chewiness were slightly reduced during fermentation, but no significant difference between 0 h and 12 h of fermentation occurred. Gumminess was also related to the gel network formed by amylose. During the fermentation period from 2 h to 12 h, it dropped dramatically from 24.0 to 19.6 g. Festus et al. [53] and Majzoobi et al. [27] also observed a decrease in gumminess after fermentation and direct treatment with organic acid. According to the analysis of variance, no significant changes were observed in the adhesiveness, resilience, cohesiveness and springiness with an increase in fermentation time.

## 4. Conclusions

In this study, the structural, thermal and physico-chemical properties of wheat starch during bifidobacteria fermentation were investigated. With the increased fermentation time, starch granules exhibited obviously corroded voids and were gradually damaged in large A-type starch granules. The solubility and swelling power were increased by fermentation. Crystallinity was also increased without altering the starch crystalline structure, indicating the destruction in the amorphous regions. The peak temperatures of gelatinization and the enthalpy of gelatinization were increased by fermentation, while setback values were decreased. The decrease in setback values indicated that the tendency of starch to retrograde was improved significantly, which is desirable for extending the shelf life of fermented products. This research indicated that bifidobacteria fermentation was an effective method for the modification of the structure and physico-chemical properties of wheat starch for further applications.

## Figures and Tables

**Table 1:** Thermal properties of wheat starch separated from bifidobacteria-fermented dough at different fermentation times.

Sample	To/°C	Tp/°C	Tc/°C	Tc-To/°C	∆H/(J/g)
Ferm-0 h	57.35 ± 0.76 ^a^	62.06 ± 0.06 ^b^	69.03 ± 0.25 ^a^	11.68 ± 1.00 ^a^	6.77 ± 0.06 ^b^
Ferm-2 h	58.92 ± 0.24 ^a^	63.86 ± 0.02 ^a^	71.09 ± 0.81 ^a^	12.17 ± 1.05 ^a^	8.26 ± 0.01a ^b^
Ferm-6 h	57.89 ± 1.20 ^a^	62.96 ± 0.88 ^ab^	70.58 ± 2.28 ^a^	12.69 ± 1.08 ^a^	9.30 ± 0.39 ^a^
Ferm-9 h	58.98 ± 0.13 ^a^	63.75 ± 0.08 ^a^	70.66 ± 2.65 ^a^	11.68 ± 2.79 ^a^	7.26 ± 1.52 ^b^
Ferm-12 h	58.80 ± 0.20 ^a^	63.52 ± 0.07 ^a^	69.68 ± 0.34 ^a^	10.88 ± 0.54 ^a^	7.56 ± 0.03 ^ab^

Results are means ± standard deviation. Data in the same column with different letters are significantly different (*p* < 0.05).

**Table 2:** Pasting properties of wheat starch separated from bifidobacteria-fermented dough at different fermentation times.

Sample	Peak Viscosity/cp	Trough/cP	Breakdown/cP	Final Viscosity/cP	Setback/cP
Ferm-0 h	2247 ± 12.72 ^a^	1769.5 ± 27.58 ^a^	477.5 ± 40.30 ^a^	2816.5 ± 2.12 ^a^	1047 ± 29.69 ^a^
Ferm-2 h	2033 ± 32.53 ^b^	1512.5 ± 13.43 ^b^	520.5 ± 45.96 ^a^	2221 ± 14.14 ^c^	708.5 ± 0.71 ^d^
Ferm-6 h	2242.5 ± 28.99 ^a^	1735.5 ± 120.92 ^a^	507 ± 149.91 ^a^	2539.5 ± 101.12 ^b^	804 ± 19.79 ^c^
Ferm-9 h	2221 ± 66.47 ^a^	1653 ± 31.11 ^ab^	568 ± 35.35 ^a^	2530 ± 41.01 ^b^	877 ± 9.89 ^b^
Ferm-12 h	2168.5 ± 10.61 ^a^	1679.5 ± 45.96 ^a^	489 ± 35.35 ^a^	2413.5 ± 33.23 ^b^	734 ± 12.73 ^d^

Results are means ± standard deviation. Data in the same column with different letters are significantly different (*p* < 0.05).

**Table 3:** Gel textural properties of wheat starch separated from bifidobacteria-fermented dough at different fermentation times.

Sample	Hardness/g	Adhesiveness/g·s	Resilience	Cohesiveness	Springiness	Gumminess/g	Chewiness/g
Ferm-0 h	50.1 ± 2.7 ^a^	96.1 ± 7.0 ^a^	5.9 ± 1.286 ^a^	0.5 ± 0.0 ^a^	92.6 ± 0.0 ^a^	22.9 ± 2.2 ^ab^	21.2 ± 2.0 ^ab^
Ferm-2 h	52.2 ± 1.9 ^a^	91.9 ± 35.8 ^a^	3.9 ± 1.149 ^a^	0.5 ± 0.0 ^a^	92.9 ± 1.8 ^a^	24.0 ± 0.2 ^a^	22.3 ± 0.6 ^a^
Ferm-6 h	48.6 ± 0.9 ^ab^	78.6 ± 19.8 ^a^	5.0 ± 0.635 ^a^	0.5 ± 0.0 ^a^	92.9 ± 1.4 ^a^	22.0 ± 0.1 ^ab^	20.4 ± 0.2 ^ab^
Ferm-9 h	45.0 ± 0.8 ^b^	77.9 ± 0.1 ^a^	4.3 ± 0.387 ^a^	0.5 ± 0.0 ^a^	92.4 ± 0.6 ^a^	22.7 ± 0.8 ^ab^	21.0 ± 0.8 ^ab^
Ferm-12 h	38.7 ± 1.2 ^c^	77.3 ± 18.9 ^a^	5.1 ± 1.688 ^a^	0.5 ± 0.0 ^a^	92.6 ± 2.3 ^a^	19.6 ± 1.7 ^b^	18.1 ± 1.1 ^b^

Results are means ± standard deviation. Data in the same column with different letters are significantly different (*p* < 0.05).

## Data Availability

The data presented in this study are available in this article.
